# Characteristic Analysis of *Ictalurus punctatus STING* and Screening Validation of Interacting Proteins with *Ictalurid herpesvirus* 1

**DOI:** 10.3390/microorganisms13081780

**Published:** 2025-07-30

**Authors:** Lihui Meng, Shuxin Li, Hongxun Chen, Sheng Yuan, Zhe Zhao

**Affiliations:** 1 Laboratory of Aquatic Parasitology and Microbial Bioresources, School of Marine Science and Engineering, Qingdao Agricultural University, Qingdao 266237, China; lihuimeng@qau.edu.cn; 2 Jiangsu Province Engineering Research Center for Marine Bio-Resources Sustainable Utilization, College of Oceanography, Hohai University, No. 1 Xikang Road, Nanjing 210098, China; lishuxin@hhu.edu.cn (S.L.); chenhongxun@hhu.edu.cn (H.C.); 210211080007@hhu.edu.cn (S.Y.); 3 School of Aquatic Science and Technology, Jiangsu Agri-Animal Husbandry Vocational College, Taizhou 225300, China

**Keywords:** *IpSTING*, Type I IFN, CCV, yeast-two-hybrid, ORF41, ORF65

## Abstract

The innate immune response is an important defense against invading pathogens. Stimulator of interferon gene (STING) plays an important role in the cyclic GMP-AMP synthase (cGAS)-mediated activation of type I IFN responses. However, some viruses have evolved the ability to inhibit the function of STING and evade the host antiviral defenses. Understanding both the mechanism of action and the viruses targets of STING effector is important because of their importance to evade the host antiviral defenses. In this study, the STING (*IpSTING*) of *Ictalurus punctatus* was first identified and characterized. Subsequently, the yeast two-hybrid system (Y2HS) was used to screen for proteins from channel catfish virus (CCV, *Ictalurid herpesvirus* 1) that interact with IpSTING. The ORFs of the CCV were cloned into the pGBKT7 vector and expressed in the AH109 yeast strain. The bait protein expression was validated by autoactivation, and toxicity investigation compared with control (AH109 yeast strain transformed with empty pGBKT7 and pGADT7 vector). Two positive candidate proteins, ORF41 and ORF65, were identified through Y2HS screening as interacting with IpSTING. Their interactions were further validated using co-immunoprecipitation (Co-IP). This represented the first identification of interactions between IpSTING and the CCV proteins ORF41 and ORF65. The data advanced our understanding of the functions of ORF41 and ORF65 and suggested that they might contribute to the evasion of host antiviral defenses. However, the interaction mechanism between IpSTING, and CCV proteins ORF41 and ORF65 still needs to be further explored.

## 1. Introduction

The channel catfish (*Ictalurus punctatus*), a member of the family *Amblycipitidae* in the order *Siluriformes*, is a freshwater species of significant economic value in China. However, outbreaks of viral diseases, particularly channel catfish virus (CCV, *Ictalurid herpesvirus 1*), the type species of the genus *Ictalurivirus* in the family *Alloherpesviridae*, have led to substantial economic losses in the channel catfish aquaculture industry [1]. Given that teleosts predominantly rely on innate immunity as their primary defense mechanism, investigating these responses is of both scientific and economic importance for developing effective antiviral strategies. Viral infections activate innate immune systems, triggering early host defense responses, such as the induction of interferons (IFNs) [2]. Recent studies have highlighted the critical role of the stimulator of interferon genes (*STINGs*) in recognizing cytoplasmic DNA and activating innate immunity against various DNA and RNA viruses [3,4,5]. STING has been identified as a transmembrane component of the endoplasmic reticulum (ER), crucial for producing type I IFN in response to cytoplasmic double-stranded DNA (dsDNA), DNA and RNA viruses, as well as intracellular bacteria in the fibroblasts, macrophages, and dendritic cells (DCs) [6,7].

Recent studies have highlighted the involvement of STING in innate immunity in certain aquatic vertebrates. The STING protein was first discovered in crucian carp (*Carassius carassius*), where it was shown to inhibit viral replication via the IFN response pathway dependent on IRF3/7 [8]. STING orthologs in zebrafish (*Danio rerio*) and fathead minnow (*Pimephales promelas*) function similarly to mammalian STING-inducing IFN and ISGs to protect against Rhabdovirus and Epizootic Hematopoietic Necrosis Virus [9]. In grass carp (*Ctenopharyngodon idella*), STING triggers IFN production in response to poly(I:C), and mediates immune responses to viral or bacterial PAMPs via an IFN-independent pathway [10]. To date, the severity of epizootic channel catfish virus disease (CCVD) has escalated considerably, leading to substantial economic losses and threatening the sustainable development of the channel catfish industry [1]. However, the precise role of STING in antiviral immune responses in channel catfish remains unclear.

In this study, the STING (*IpSTING*) was identified and characterized in the channel catfish (*Ictalurus punctatus*) through bioinformatics analysis. Subsequently, CCV proteins ORF41 and ORF65, which interacted with IpSTING, were identified through a yeast two-hybrid system, and their interactions were further confirmed by co-immunoprecipitation (Co-IP). This study provided a foundation for further exploration of the role of STING in the innate immunity of aquatic species. However, the specific mechanism of IpSTING in the response to CCV infection in channel catfish remains to be further investigated.

## 2. Materials and Methods

### 2.1. Cell and Virus

Channel catfish ovary (CCO) cells were cultured in Eagle’s medium modified by Dulbecco (DMEM, Gibco, Waltham, MA, USA), supplemented with 10% fetal bovine serum (FBS, Gibco, USA), 100 IU/mL penicillin, and 0.1 mg/mL streptomycin (Sigma, St. Louis, MO, USA), and maintained at 28 °C. The CCV (strain VR-665) was kindly provided by Prof. Jun-Fa Yuan of Huazhong Agricultural University, and was propagated in CCO cells cultured in DMEM supplemented with 10% FBS until a cytopathic effect (CPE) was observed.

20 mL of virus supernatant was collected and cell debris was removed by gradient centrifugation at 8000× *g* and 12,000× *g* with 4 °C for 30 min. The supernatant was then filtered through a 0.22 μm filter to further eliminate cellular components. Subsequently, the CCV-containing supernatant was concentrated by centrifugation at 4000 × *g* using an Amicon Ultra column (molecular weight cutoff: 100 kDa; Millipore, Burlington, MA, USA). The purified virus was then harvested and stored at −80 °C for future analyses.

HEK-293T cells (human embryonic kidney cells, 293T subline) were cultured in DMEM supplemented with 10% FBS and 1% penicillin-streptomycin solution (Pen-Strep, containing 100 U/mL penicillin and 100 μg/mL streptomycin). The culture conditions were maintained at 37 °C in a humidified incubator with 5% CO_2_.

### 2.2. RNA Extraction and cDNA Synthesis

Total RNA was isolated from CCO cells using Trizol Reagent (Invitrogen, Waltham, MA, USA) according to the manufacturer’s instructions, followed by treatment with RNase-free DNase I (TaKaRa, Beijing, China) to remove genomic DNA contamination. The concentration and integrity of the RNA were assessed by 1.5% agarose gel electrophoresis and spectrophotometry. cDNA was synthesized from 1 μg of RNA using a Reverse Transcriptase M-MLV Kit (TaKaRa, Beijing, China) following the manufacturer’s protocol and stored at −20 °C.

### 2.3. Gene Cloning and Plasmid Construction

The open reading frame (ORF) of IpSTING (GenBank accession no. XP_053541768.1) was amplified from channel catfish cDNA using gene-specific primers (Table 1). PCR amplification was carried out using Premix Taq (Takara, China) with an initial denaturation at 98 °C for 3 min, followed by 35 cycles of 98 °C for 10 s, 55 °C for 5 s, and 72 °C for 10 s. A final extension was performed at 72 °C for 10 min. Amplified products were analyzed by 1.2% agarose gel electrophoresis and purified using a PCR Purification Kit (OMEGA, Norcross, GA, USA).

In parallel, the coding regions of 73 ORFs from the channel catfish virus (CCV) genome were amplified using genomic DNA extracted with the TIANGEN Virus DNA/RNA Kit (TIANGEN, Nanjing, China). Each ORF was individually cloned into the pGBKT7 vector using a One Step Cloning Kit (Vazyme, Nanjing, China) to construct bait plasmids for yeast two-hybrid system (Y2HS) screening.

For prey plasmid construction, the IpSTING coding sequence was cloned into the pGADT7 vector (AD-IpSTING). To validate protein–protein interactions identified in Y2HS, IpSTING was also cloned into the pCMV-C-Myc vector, while selected viral genes (ORF41 and ORF65) were inserted into the pcDNA3.1/His vector for co-expression in CCO cells. All constructs were verified by Sanger sequencing following transformation into *E. coli DH5α*. Details of plasmid constructs and their applications are summarized in Table 2.

### 2.4. Sequence Analysis of IpSTING

The protein sequence of IpSTING was deduced using the ExPASy Translate Tool (https://web.expasy.org/translate/, accessed on 20 April 2025). Protein similarity was analyzed using BLASTP (v2.8) with NCBI nr-database. Protein characteristics, including molecular weight, amino acid composition, and theoretical isoelectric point (pI), were analyzed using the ProtParam tool (https://web.expasy.org/protparam/, accessed on 20 April 2025). Conserved domains were predicted using the SMART program (http://smart.emblheidelberg.de/, accessed on 20 April 2025). Signal peptides were predicted using SignalP 5.0 (http://www.cbs.dtu.dk/services/SignalP/, accessed on 20 April 2025). The secondary structure construction was performed using NOVOPRO (https://novopro.cn/tools/secondary-structure-prediction.html, accessed on 20 April 2025). Multiple sequence alignment was performed using the Clustal Omega (1.2.4) tool available on the EBI website (https://www.ebi.ac.uk/Tools/msa/clustalo/, accessed on 20 April 2025). Phylogenetic analysis of STING was conducted using the neighbor-joining (NJ) method in MEGA 7.0 software with 1000 bootstrap repeats.

### 2.5. Yeast Reporter Assay

First, the potential toxicity and autoactivation of the bait protein were evaluated. The pGBKT7-53/pGADT7-T were used as positive controls, while pGBKT7-Lam/pGADT7-T served as negative controls. AH109 yeast cells were co-transformed with pGBKT7-ORF and pGADT7. The transformants were grown on SD/-Leu/-Trp (DDO) agar plates for 3 to 5 days. Colonies were then transferred to SD/-Ade/-His/-Leu/-Trp/X-α-Gal (QDO/X) plates and incubated for 4 to 7 days. The absence of blue colonies on QDO/X plates indicated that the bait protein did not exhibit autoactivation. The bait protein, which lacked autoactivation, was then co-transformed with pGADT7-IpSTING into AH109 yeast cells.

### 2.6. Transient Transfection Assay

Plasmids were transfected into HEK-293T cells using Lipofectamine 2000 (Invitrogen, Waltham, MA, USA) according to the manufacturer’s instructions. The cultured cells were seeded into 6-well plates at a density of 4.5 × 10^5^ adherent cells per well. The cells were then co-transfected with 2.5 µg of pCMV-C-Myc-IpSTING (IpSTING-Myc) and either pcDNA3.1-His, pcDNA3.1-ORF41 (ORF41-His), or pcDNA3.1-ORF65 (ORF65-His). Simultaneously, 2.5 µg of pCMV-C-Myc was co-transfected with pcDNA3.1-ORF41 or pcDNA3.1-ORF65, respectively. Given that protein expression in CCO cells peaked at 48 h post-transfection, cells were collected at same time point for Co-IP analysis.

### 2.7. Co-IP Analysis and Western Blot

The cell lysates were harvested by centrifugation (Thermo Fisher, Waltham, MA, USA) at 12,000 rpm for 25 min at 4 °C, and subsequently incubated overnight at 4 °C with Flag-tagged antibody (CST, Danvers, MA, USA). Protein A/G Plus-Agarose (CST, USA) was then added, and the mixture was incubated at 4 °C for 6 h. The immunoprecipitates were collected by centrifugation at 800 rpm for 3 min at 4 °C, washed three times with ice-cold phosphate-buffered saline (PBS), and eluted in 50 μL of PBS. The resulting sample was designated as the immunoprecipitation (IP) sample for interaction detection via Western blotting. Briefly, the proteins were transferred onto polyvinylidene fluoride (PVDF) membrane (Millipore, Burlington, MA, USA) and blocked with 5% skim milk (Sangon, Shanghai, China). The blocked membranes were then incubated with primary antibodies (mouse anti-His-Tag mAbs, 2366T; mouse anti-Myc-Tag mAbs, 2276, CST, USA) and secondary antibodies (HRP-labeled goat anti-mouse IgG, A0216, Beyotime, Shanghai, China). The Pierce ECL Plus Western Blotting Substrate (Vazyme, Nanjing, China) was used for signal detection.

## 3. Result

### 3.1. Sequences Analysis of IpSTING

**Figure 1:**
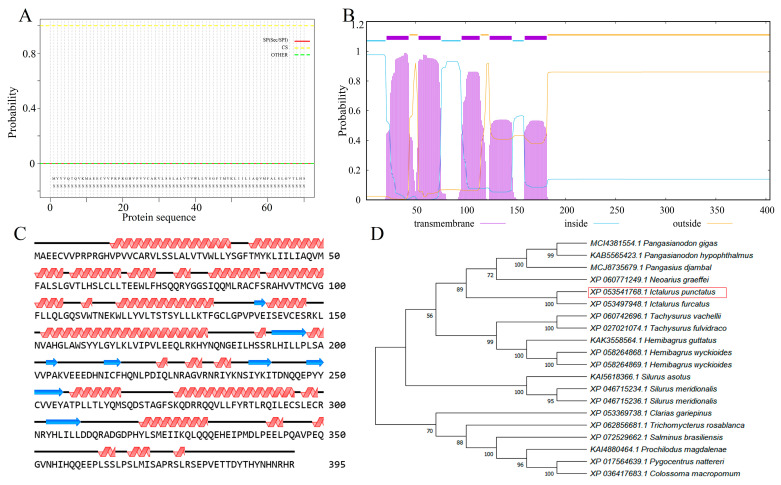
Structural characteristics and evolutionary analysis of IpSTING protein sequence. (**A**) Prediction and analysis of signal peptide structure. (**B**) Prediction and analysis of transmembrane structures. (**C**) Secondary structure prediction analysis. (**D**) Phylogenetic tree inferred for STING using the neighbor-joining method in MEGA 7.0. Bootstrapping with 1000 replicates is shown at nodes. The analysis includes 20 amino acid sequences, scale bar (0.2) represents genetic distance. The red box in the figure represents IpSTING in this study.

The cDNA sequence of STING was 1185 base pairs (bp), encoding a protein of 395 amino acids, with a predicted isoelectric pI of 6.41 and a molecular weight of 45.61 kDa (Supplementary Figure S1). The IpSTING protein lacked signal peptides (Figure 1A), but contained five transmembrane regions located at positions 12–34AA, 44–66AA, 87–105AA, 115–137AA, and 150–172AA (Figure 1B). The secondary structure of IpSTING protein contained 25 alpha helices and 8 beta folds (Figure 1C). In the phylogenetic analysis, IpSTING clustered closely with the STING plumieribetin-like sequence from *Ictalurus furcatus*, forming a distinct clade with other fish species (Figure 1D).

**Figure 2:**
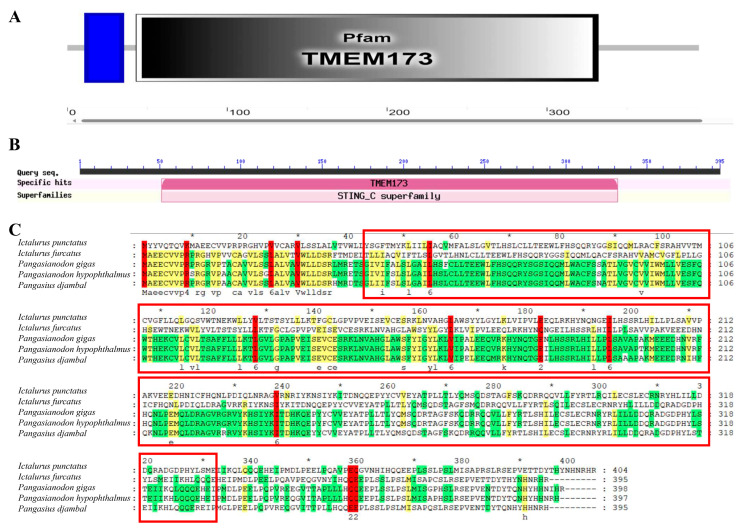
Multi sequence alignment analysis of conserved domains of IpSTING protein. (**A**) The conserved domains of the IpSTING protein amino acid sequence are predicted using the SMART program. (**B**) The conserved domains of the IpSTING protein amino acid sequence are predicted using the NCBI BLASTP (v2.8). (**C**) Homology alignment of IpSTING with STING sequences from other species. Identical amino acids are shaded in red, the red box indicates the conserved domain of TMEM173, and an asterisk (*) was marked every 20 amino acids.

Conservative functional domain analysis showed that IpSTING harbored a conserved domain (residues 43–332AA), corresponding to Transmembrane Protein 173 (TMEM173) (Figure 2A,B). TMEM173, also known as stimulator of interferon genes protein (STINGs) or endoplasmic reticulum interferon stimulator (ERIS), was a transmembrane adaptor protein involved in innate immune signaling. It could induce the expression of type I interferons (IFN-alpha and IFN-beta) through the NF-kappa-B and IRF3 pathways in response to non-self-cytosolic RNA and dsDNA. Multiple sequence alignments indicated that the IpSTING sequences of STING and *Ictalurus furcatus* exhibited the highest sequence similarity (Figure 2C).

### 3.2. Autoactivation and Toxicity Tests of pGBKT7-ORF Bait

**Figure 3:**
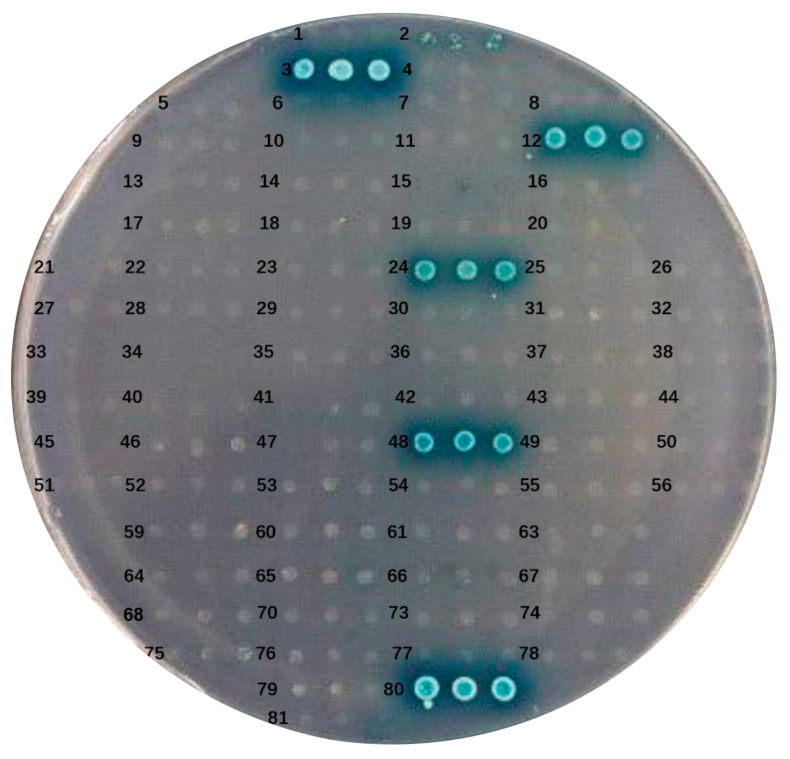
Autoactivation and toxicity tests of pGBKT7-ORF plasmid. Numbers 1–79 colonies represent the self-activation analysis of the bait plasmid ORF1-ORF79: pGBKT7-ORF and pGADT7-T, respectively. Colony number 80 represents a positive control group: pGBKT7-53 and pGADT7-T. Colony number 81 represents a negative control group: pGBKT7-Lam and pGADT7-T.

To evaluate the autoactivation capability of the bait protein in yeast cells, pGBKT7-ORF and the pGADT7 empty plasmid were co-transformed into AH109 cells. The resulting transformants were cultured on DDO and QDO/X media. The co-transformed AH109 yeast cells exhibited robust growth on DDO, indicating successful co-transformation, and no adverse effects on yeast growth due to the ORF-expressed protein were observed. Autoactivation from the bait protein would lead to the expression of reporter genes, resulting in the formation of blue colonies on QDO/X plates. Yeast cells co-transformed with pGBKT7-ORF3, pGBKT7-ORF12, pGBKT7-ORF24, pGBKT7-ORF48, and the pGADT7 empty plasmid were able to grow and exhibited a blue color on QDO/X/A plates (Figure 3). These results demonstrated the strong transcriptional autoactivation activity of ORF3, ORF12, ORF24, and ORF48, while the remaining 69 ORFs showed no autoactivation activity.

### 3.3. The ORF41 and ORF65 of CCV Potentially Interact with the IpSTING

**Figure 4:**
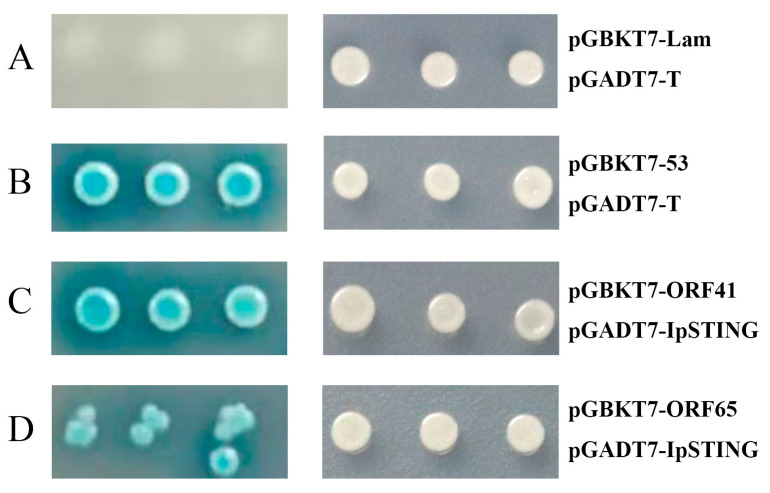
Interaction validation analyses of pGADT7-IpSTING with candidate CCV proteins using the yeast two-hybrid system (Y2HS). (**A**–**D**) show representative images of yeast AH109 transformants grown on DDO (right) and QDO/X (left) plates. (**A**) Negative control: pGBKT7-Lam + pGADT7-T. (**B**) Positive control: pGBKT7-53 + pGADT7-T. (**C**) Test group: pGBKT7-ORF41 + pGADT7-IpSTING. (**D**) Test group: pGBKT7-ORF65 + pGADT7-IpSTING. Growth and blue coloration on QDO/X plates indicate a positive interaction.

The plasmid pGADT7-IpSTING and 69 pGBKT7-ORFs were co-transformed into AH109 cells and incubated on DDO and QDO/X media, respectively. Co-transformation of pGBKT7-53/pGADT7-T served as the positive control, while pGBKT7-Lam/pGADT7-T was used as the negative control. The positive control showed blue colonies, whereas the negative control showed no blue colonies on QDO/X plates (Figure 4). Protein interactions were assessed by comparing the growth and color of the yeast with the positive and negative controls. The AH109 yeast strain, harboring pGBKT7-ORF41 or pGBKT7-ORF65 and pGADT7-IpSTING, grew well on medium lacking tryptophan and leucine and formed distinct blue colonies on QDO/X medium. However, under the same screening conditions, no blue colonies were detected in the combination of pGADT7-IpSTING co-transformed with all 67 other pGBKT7 ORFs. Thus, the interaction between IpSTING and ORF41 and ORF65 from CCV was confirmed (Figure 4).

### 3.4. Identification of the Interaction Between ORF41 or ORF65 and IpSTING by Co-IP

**Figure 5:**
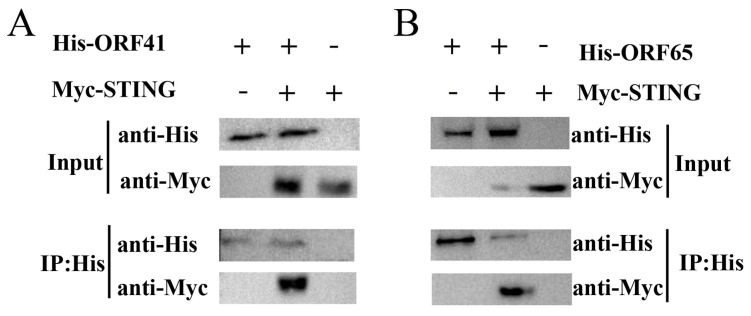
Co-IP assay analysis of the interaction between IpSTING and ORF41 or ORF65. (**A**) Immunoprecipitation of IpSTING and ORF41. HEK-293T cells were co-transfected with the expression plasmids for His-tagged ORF41 and Myc-tagged IpSTING. Cell lysates were prepared 48 h after transfection. Input protein samples and samples immunoprecipitated with either anti-His or anti-Myc antibodies were separated on SDS-PAGE gels and analyzed with anti-His and anti-Flag antibodies, respectively. Negative control was analyzed simultaneously. (**B**) Immunoprecipitation of IpSTING and ORF65.

To investigate the interaction between ORF41 or ORF65 and IpSTING, we co-transfected the vectors pCMV-C-Myc-IpSTING and either pcDNA3.1-ORF41 or pcDNA3.1-ORF65 into HEK-293T cells. In HEK-293T cells, Co-IP assays were performed using an anti-His antibody as bait, and immunoblotting revealed a STING band in cells co-expressing ORF41-His and IpSTING-Myc (Figure 5A). Similarly, immunoblotting detected a IpSTING band in HEK-293T cells co-expressing ORF65-His and IpSTING-Myc (Figure 5B). These Co-IP results demonstrate that ORF41 and ORF65 interact with IpSTING.

## 4. Discussion

CCV, a significant member of the family *Alloherpesviridae*, is known for causing lethal hemorrhagic infections in juvenile channel catfish [11]. The devastating impact of CCV on catfish fingerlings has been well-documented, leading to substantial economic losses [12,13]. Currently, research primarily focuses on antiviral strategies [14] and gene functions [13,15], with relatively less attention given to the interaction mechanisms between CCV and channel catfish. Notably, STING has been identified as a critical mediator of the innate immune response to cytosolic DNA and RNA from diverse pathogens, yet its specific role in CCV infection remains largely unexplored [16,17]. Additionally, STING has been shown to play a significant role in pathogen infection in mammals. For instance, overexpression of STING has been observed to reduce the intracellular viral loads during Japanese encephalitis virus infection [18]. Additionally, STING-deficient mice exhibit increased susceptibility to lethal infection following exposure to HSV-1. Moreover, knockdown of STING impairs the production of type I IFN in HEK293 and HeLa cells following infection with Sendai virus and Vesicular stomatitis virus [19]. The overexpression of STING has shown protection against RNA virus infections, significantly inhibiting the replication of both DNA and RNA viruses in zebrafish cells [9]. Similarly, STING expression in crucian carp, grass carp, grouper, and black carp has been observed to significantly inhibit viral replication in vivo by activating an IRF3/7-dependent IFN response [8,10,20,21].

STING plays a crucial role in the activation of type I IFN in response to DNA pathogens. In mammals, several upstream factors, including cyclic GMP-AMP synthase (cGAS), helicase DDX41, DNA-binding protein DAI, and the IFN-inducible sensor IFI16, contribute to the antiviral response by activating STING and promoting TBK1-dependent phosphorylation of IRF3 [22,23,24,25,26]. Among these, cGAS has been identified as the primary sensor in the STING signaling pathway. Recent studies have reported the involvement of cGAS and DDX41 in the innate immunity of certain aquatic vertebrates. However, direct evidence demonstrating that these DNA sensors function through STING in fish is still lacking. Additionally, mammalian STING functions as a pattern recognition receptor (PRR) by binding to cyclic dinucleotides, such as cyclic di-GMP, cyclic di-AMP, and cyclic GAMP, as well as dsDNA and ssDNA, to initiate the IFN response [27,28,29,30,31]. However, it remains unclear whether fish STING directly recognizes DNA or cyclic dinucleotides [20,21]. Therefore, further investigation is needed to elucidate the detailed mechanisms of STING in fish innate immunity.

The Y2HS has proven to be an effective method for verifying protein interactions [32] and for screening unknown proteins that interact with known proteins. Due to its simplicity and high screening efficiency, it has become a widely used method for protein interaction screening [33]. The specific interaction mechanism between CCV and STING from channel catfish remains undetermined. In this study, we successfully identified and characterized the STING in *Ictalurus punctatus* and identified that the encoded protein was a membrane protein. Next, we identified ORF41 and ORF65 as proteins that interact with the IpSTING protein of channel catfish using Y2HS screening. These findings supported the potential role of STING as a DNA sensor and its involvement in activating host immune responses [4]. To validate the Y2HS results and exclude false-positive interactions, Co-IP assays were further performed. The Co-IP assay results clearly demonstrated the specific interaction between IpSTING and ORF41 or ORF65. Therefore, it was proposed that ORF41 and ORF65 were putative interaction partners of IpSTING in CCV. These findings significantly enhanced our understanding of the antiviral mechanism of IpSTING and suggested the potential use of IpSTING as a binding protein for comprehensive antiviral management. Furthermore, considering IpSTING’s role as a critical component of the cGAS-STING immune pathway, we speculated that ORF41 and ORF65 might modulate the host cGAS-STING immune pathway through their interaction with IpSTING. However, the exact mechanisms by which ORF41 and ORF65 influenced the IpSTING-mediated cGAS-STING immune pathway remained unclear and required further investigation.

In addition to ORF41 and ORF65, glycoprotein ORF59 also warrants attention [34]. ORF59 is a late-stage, membrane-associated structural protein previously confirmed to play a critical role in CCV virion assembly, membrane localization, and inhibition of viral adsorption. Notably, recombinant ORF59 has been shown to block viral entry and reduce production of infectious particles in CCO cells [35]. These features suggest that ORF59, like other herpesviral envelope glycoproteins, may engage in molecular cross-talk with host signaling proteins. Given its membrane localization and timing of expression, ORF59 is well-positioned to interface with host pattern recognition receptors such as IpSTING. Although current evidence focuses on ORF59’s structural and entry functions, it is reasonable to speculate that ORF59 may influence STING-mediated signaling pathways—possibly extending beyond classical IFN responses to include cellular stress reactions like autophagy, apoptosis, or metabolic reprogramming. Future studies using Co-IP, IRF3/NF-κB reporter assays, and cell stress response markers will help clarify whether ORF59 participates in modulating host immunity during CCV infection.

Collectively, these findings underscore the multifaceted strategies employed by CCV to interact with and potentially modulate host immune responses. By identifying STING as a key immune component in *Ictalurus punctatus* and uncovering its interaction with multiple viral proteins—including ORF41 and ORF65—this study highlights the complex interplay between CCV and the host cGAS-STING signaling axis. These insights not only enhance our understanding of CCV pathogenesis but also lay the groundwork for future antiviral strategies that target viral proteins involved in immune evasion and host modulation.

## 5. Conclusions

In conclusion, we successfully identified and characterized the STING in *Ictalurus punctatus*, as well as two CCV proteins that interacted with STING. This is the first report of a CCV protein interacting with STING. The findings suggested that STING, as a host protein, could interact with ORF41 or ORF65 of CCV, indicating a potential key role for STING in the innate immunity of channel catfish. However, further research is necessary to determine whether STING regulates type I IFN levels and to elucidate the underlying mechanisms by which it interacts with ORF41 or ORF65, thereby modulating the host’s antiviral immune response to CCV.

## Figures and Tables

**Table 1:** Primers used in this study.

Primer	Sequence (5′–3′)
AD-IpSTING-F	gtaccagattacgctcatatgATGGCGGAGGAGTGTGTGG
AD-IpSTING-R	acgattcatctgcagctcgagTTATCTGTGTCTGTTGTGGTTATAGTGG
BD-ORF1-F	tcagaggaggacctgcatatgATGGACGGTCTGAAGGAAATCA
BD-ORF1-R	ttcggcctccatggccatatgTCAGGATAGCAGAAGGGTTTTTG
BD-ORF2-F	tcagaggaggacctgcatatgATGACAGGTAGGCACTCGCCG
BD-ORF2-R	ttcggcctccatggccatatgTCAGAGAGGTAAGAGTGGAGCATG
BD-ORF3-F	tcagaggaggacctgcatatgATGGCATTCTCCACGTCTGACG
BD-ORF3-R	ttcggcctccatggccatatgCTAGTTGAGGAGCGCACGTCG
BD-ORF4-F	tcagaggaggacctgcatatgATGGCGTCCATCGATGTGG
BD-ORF4-R	ttcggcctccatggccatatgTCAGAGCCAGTCCTGTCGATCG
BD-ORF5-F	tcagaggaggacctgcatatgATGGCGCTCAGGGAAGGT
BD-ORF5-R	ttcggcctccatggccatatgTTATAACTGACTTTTTCGTGTTAGGGG
BD-ORF6-F	tcagaggaggacctgcatatgATGAACTCTCTCACTATCATCTTCCTCC
BD-ORF6-R	ttcggcctccatggccatatgTCAGACCCGGATCTCCGC
BD-ORF7-F	tcagaggaggacctgcatatgATGGCCGCCGTCATATTAGA
BD-ORF7-R	ttcggcctccatggccatatgTCACAGGGAGTAATCGATGCG
BD-ORF8-F	tcagaggaggacctgcatatgATGAAATGTCCGCGCGCA
BD-ORF8-R	ttcggcctccatggccatatgTTATAGACCGGGGCTCCAGTC
BD-ORF9-F	tcagaggaggacctgcatatgATGGCAACCAGACCCAAGG
BD-ORF9-R	ttcggcctccatggccatatgTCACGGCGCGCAGGTGCA
BD-ORF10-F	tcagaggaggacctgcatatgATGACCGTCAAGGGTTGCC
BD-ORF10-R	ttcggcctccatggccatatgTTAGATGTACGGAGACATCATTGTCG
BD-ORF11-F	tcagaggaggacctgcatatgATGAGGTGTATTCGAGCAGCG
BD-ORF11-R	ttcggcctccatggccatatgTCAGTGGCCTGTTGGTGGAT
BD-ORF12-F	tcagaggaggacctgcatatgATGACAAGCCCCCGCGAG
BD-ORF12-R	ttcggcctccatggccatatgTCAGCGCGAACAGGCATC
BD-ORF13-F	tcagaggaggacctgcatatgATGGCGTTTCTATTGCCCTTC
BD-ORF13-R	ttcggcctccatggccatatgTCACCTGAGACCGTCCACGG
BD-ORF14-F	tcagaggaggacctgcatatgATGGCGGAGAAGTTGATCCC
BD-ORF14-R	ttcggcctccatggccatatgTTATATCTTGGAGAATTCGTCCACG
BD-ORF15-F	tcagaggaggacctgcatatgATGGCAGCGGTTAATTGGC
BD-ORF15-R	ttcggcctccatggccatatgTCAATGTTCGGGTTGGGTTG
BD-ORF16-F	tcagaggaggacctgcatatgATGACATCTTCGGGATTCCTCA
BD-ORF16-R	ttcggcctccatggccatatgTCACAATTTAAACTTTTCGATAAGCA
BD-ORF17-F	tcagaggaggacctgcatatgATGATCCCGCCCGGGATC
BD-ORF17-R	ttcggcctccatggccatatgCTACACGGTAGACCCGGGTTC
BD-ORF18-F	tcagaggaggacctgcatatgATGAAAGCGTCCCAAGAGCG
BD-ORF18-R	ttcggcctccatggccatatgTTATTCATCATCATCATCACCACCA
BD-ORF19-F	tcagaggaggacctgcatatgATGGAAACAATGGGGCCCA
BD-ORF19-R	ttcggcctccatggccatatgTTACTCGTTCGCGATAGTGTACG
BD-ORF20-F	tcagaggaggacctgcatatgATGGCGTCCTCGGATAGGTT
BD-ORF20-R	ttcggcctccatggccatatgTCACGATTCAGGATCAACAAGAAA
BD-ORF21-F	tcagaggaggacctgcatatgATGTCTGCGACACAAGGTCCA
BD-ORF21-R	ttcggcctccatggccatatgCTACTCGGACTCGGACTCGGA
BD-ORF22-F	tcagaggaggacctgcatatgATGGGTCCCATACGGAAGCC
BD-ORF22-R	ttcggcctccatggccatatgTTATTCAGAGTCTGAACCAGTGACACT
BD-ORF23-F	tcagaggaggacctgcatatgATGTACATCCGTATTAATGAGTCCCG
BD-ORF23-R	ttcggcctccatggccatatgTCACCAAAGGTTCCGAGGTTT
BD-ORF24-F	tcagaggaggacctgcatatgATGACGTCCTCCATACCCGC
BD-ORF24-R	ttcggcctccatggccatatgTCACTCGACGCACGCGGT
BD-ORF25-F	tcagaggaggacctgcatatgATGGACGTTGAGATCAAAGAGTATGC
BD-ORF25-R	ttcggcctccatggccatatgTCAATAAGGACATTCTCTCGGGA
BD-ORF26-F	tcagaggaggacctgcatatgATGCAACTCGTTCGCGAAA
BD-ORF26-R	ttcggcctccatggccatatgTCATATGATTTTAAGTCGACACTTGTTT
BD-ORF27-F	tcagaggaggacctgcatatgATGGCGTCCATAACCGGTC
BD-ORF27-R	ttcggcctccatggccatatgTCAAACACCTGAAAATTGTTTAATGA
BD-ORF28-F	tcagaggaggacctgcatatgATGTCACTACCAGGCGGAAGAG
BD-ORF28-R	ttcggcctccatggccatatgTTACAGATTCATGAGCGTCTCGA
BD-ORF29-F	tcagaggaggacctgcatatgATGACCCGAAAAATTGACGC
BD-ORF29-R	ttcggcctccatggccatatgTCAAATTCTTGACATGATACTGTCTCG
BD-ORF30-F	tcagaggaggacctgcatatgATGATGGATATTTTCAATTCGGTG
BD-ORF30-R	ttcggcctccatggccatatgTTACAACGGTGCAGCGGTACC
BD-ORF31-F	tcagaggaggacctgcatatgATGTATTTACAAATGAATTTTTTTCCG
BD-ORF31-R	ttcggcctccatggccatatgTTATTTCCCACTCGTGTATAAAATGAC
BD-ORF32-F	tcagaggaggacctgcatatgATGAGGGGGGCGACCACG
BD-ORF32-R	ttcggcctccatggccatatgTCACGAGACAATCGCTATCGAA
BD-ORF33-F	tcagaggaggacctgcatatgATGGAGCCCATATCGTATGTCAC
BD-ORF33-R	ttcggcctccatggccatatgTTACTCATCAGGAAGCTCGCCG
BD-ORF34-F	tcagaggaggacctgcatatgATGGAACGGGTTTTGAGACAGT
BD-ORF34-R	ttcggcctccatggccatatgTCAGTCTTGATCGCCGTGATC
BD-ORF35-F	tcagaggaggacctgcatatgATGACCGCACCGATGTCTATAGA
BD-ORF35-R	ttcggcctccatggccatatgTTATTCACAGAGGTCGAGGATCTTAT
BD-ORF36-F	tcagaggaggacctgcatatgATGGACGGAATCTTCCCCAG
BD-ORF36-R	ttcggcctccatggccatatgTCAAAAGGGCCATAGGAGCG
BD-ORF37-F	tcagaggaggacctgcatatgATGTCAGGTGTTAAATTGGGGTCA
BD-ORF37-R	ttcggcctccatggccatatgTCACACGTCTTCCATCGCCA
BD-ORF38-F	tcagaggaggacctgcatatgATGTCGGTATTGGGAACGTGTT
BD-ORF38-R	ttcggcctccatggccatatgTCAAGAATTTATATCATTGATGATACCTA
BD-ORF39-F	tcagaggaggacctgcatatgATGGAAGCCATCGCACGTC
BD-ORF39-R	ttcggcctccatggccatatgTCAGACGGCGTTCAGGTCG
BD-ORF40-F	tcagaggaggacctgcatatgATGACACCAGTACCACCGAGTAGTC
BD-ORF40-R	ttcggcctccatggccatatgTTACGTGCTTCCGGACGTG
BD-ORF41-F	tcagaggaggacctgcatatgATGTATCTCAGGGATTTTCACGAGT
BD-ORF41-R	ttcggcctccatggccatatgTTAAGGCGCACACACCCTG
BD-ORF42-F	tcagaggaggacctgcatatgATGGCGTCCTTCGGAGAGC
BD-ORF42-R	ttcggcctccatggccatatgTTACATTTCCAGGAAGAGGAACG
BD-ORF43-F	tcagaggaggacctgcatatgATGGATGCACTCCGCAAGAA
BD-ORF43-R	ttcggcctccatggccatatgTTAATCCTCATCATCACCTTCGATA
BD-ORF44-F	tcagaggaggacctgcatatgATGGGCACCGCCGATGAG
BD-ORF44-R	ttcggcctccatggccatatgTCATTCTGAAACTGGAAGGTCATTG
BD-ORF45-F	tcagaggaggacctgcatatgATGACCTTCCAGTTTCAGAATGAGT
BD-ORF45-R	ttcggcctccatggccatatgTTATTTATCAGAAAACGTTTTCTCAACA
BD-ORF46-F	tcagaggaggacctgcatatgATGAAGAAAACGATGCTCGCA
BD-ORF46-R	ttcggcctccatggccatatgTCAATACATCATCCCCATCGG
BD-ORF47-F	tcagaggaggacctgcatatgATGATTGCGAGCATCGTTTTC
BD-ORF47-R	ttcggcctccatggccatatgTCACCGGATCCCGTGACA
BD-ORF48-F	tcagaggaggacctgcatatgATGAACAACGGGTGGGACG
BD-ORF48-R	ttcggcctccatggccatatgTCACCAGATAATGGGTAGTTGATCA
BD-ORF49-F	tcagaggaggacctgcatatgATGGGTGAGATGACCTCCGG
BD-ORF49-R	ttcggcctccatggccatatgTCATTGTCCTGATGAACCAAACC
BD-ORF50-F	tcagaggaggacctgcatatgATGGAATTACTTGTAGTGACCCTCAC
BD-ORF50-R	ttcggcctccatggccatatgTTACCGCCTGGCTCCACTC
BD-ORF51-F	tcagaggaggacctgcatatgATGGCCCAGTATATAGTGACGATATT
BD-ORF51-R	ttcggcctccatggccatatgTCATACTCCCCAGATAGATAGTTGCA
BD-ORF52-F	tcagaggaggacctgcatatgATGGAACAACAGAACCCGATACC
BD-ORF52-R	ttcggcctccatggccatatgTCAAAAGGATACGACTTCTCGTTTAG
BD-ORF53-F	tcagaggaggacctgcatatgATGCCCATGATGAACACCAAC
BD-ORF53-R	ttcggcctccatggccatatgCTAAACGAGAAGTCGTATCCTTTTGA
BD-ORF54-F	tcagaggaggacctgcatatgATGGGCATCAGGAAGAAGCTG
BD-ORF54-R	ttcggcctccatggccatatgTCACTTTCGACCGAGCGTG
BD-ORF55-F	tcagaggaggacctgcatatgATGTCTCTCACGAACAAACCCAG
BD-ORF55-R	ttcggcctccatggccatatgTCAGGAATCGTGGAGGGCA
BD-ORF56-F	tcagaggaggacctgcatatgATGTCGAGTGAAGTGTTCCGATG
BD-ORF56-R	ttcggcctccatggccatatgTTATCGGTTCATCAATCTCTGAATTT
BD-ORF59-F	tcagaggaggacctgcatatgATGGTCGGCAAAGGTCTCCCC
BD-ORF59-R	ttcggcctccatggccatatgTCACGCCCGGGCAGGTG
BD-ORF60-F	tcagaggaggacctgcatatgATGATGATTTTGTCAAAGGCCC
BD-ORF60-R	ttcggcctccatggccatatgTCACGTGGAACTTTTGGGC
BD-ORF61-F	tcagaggaggacctgcatatgATGATCTCCGTCGCGCGA
BD-ORF61-R	ttcggcctccatggccatatgTCAGACGAGGGCCCAAAAGTT
BD-ORF63-F	tcagaggaggacctgcatatgATGTACGTTGTCGTGTCGGTC
BD-ORF63-R	ttcggcctccatggccatatgTCACTGTCCACAGAATCCGAC
BD-ORF64-F	tcagaggaggacctgcatatgATGTCATCCCCGAGGGGG
BD-ORF64-R	ttcggcctccatggccatatgTCAGCCAATAACTCCGGTGGATA
BD-ORF65-F	tcagaggaggacctgcatatgATGGAAATAAACGCTGCAATC
BD-ORF65-R	ttcggcctccatggccatatgCTATATGATTCGCGCTTAAAAACAAT
BD-ORF66-F	tcagaggaggacctgcatatgATGGATTCCATCACGTTGA
BD-ORF66-R	ttcggcctccatggccatatgTTATTTCCATTTATCCAGCAG
BD-ORF67-F	tcagaggaggacctgcatatgATGGAATCCATTGTTTATGATAGTG
BD-ORF67-R	ttcggcctccatggccatatgTCACTGCCCTTGAGGCGT
BD-ORF68-F	tcagaggaggacctgcatatgATGTTGAGGACGATCGGTTCAC
BD-ORF68-R	ttcggcctccatggccatatgTCATCCGATGTGGACGTTCAA
BD-ORF70-F	tcagaggaggacctgcatatgATGGACGGTTACTTCAAAAATATC
BD-ORF70-R	ttcggcctccatggccatatgTTACACACAAAAATGTTTAAACAGTAAC
BD-ORF73-F	tcagaggaggacctgcatatgATGGCAGATAGGACACCCAAGC
BD-ORF73-R	ttcggcctccatggccatatgTCAACTTAGTGTCCCGCTGGC
BD-ORF74-F	tcagaggaggacctgcatatgATGAGCGGGAAGGTCGAAA
BD-ORF74-R	ttcggcctccatggccatatgTCACCGACTGCCCGTGAT
BD-ORF75-F	tcagaggaggacctgcatatgATGGTCAATACATTAAAAAGTTCCC
BD-ORF75-R	ttcggcctccatggccatatgTCATGACAGCGACTCGAGG
BD-ORF76-F	tcagaggaggacctgcatatgATGGACCGGCATCGGTAT
BD-ORF76-R	ttcggcctccatggccatatgTCACGGGAATATTCTCGGG
BD-ORF77-F	tcagaggaggacctgcatatgATGGCACGGAAAGGAGAGC
BD-ORF77-R	ttcggcctccatggccatatgTCACGGTAAGAATGTCGACACC
BD-ORF78-F	tcagaggaggacctgcatatgATGGAGTTATCACTGTACCGCG
BD-ORF78-R	ttcggcctccatggccatatgTCAGTGTGAATTATTGGAATGGG
BD-ORF79-F	tcagaggaggacctgcatatgATGGATTTCGAATGCGGTTC
BD-ORF79-R	ttcggcctccatggccatatgTCAGCCGGTCTGGACGC
pCDNA-ORF41-F	tagtccagtgtggtggaattcATGTATCTCAGGGATTTTCACGAGT
pCDNA-ORF41-R	tgctggatatctgcagaattcAGGCGCACACACCCTGACT
pCDNA-ORF65-F	tagtccagtgtggtggaattcATGGAAATAAACGCTGCAATCG
pCDNA-ORF65-R	tgctggatatctgcagaattcTATATGATTCGCGCTTAAAAACACTG
pCMV-Myc-STING-F	tccaagcttctgcaggaattcATGGCGGAGGAGTGTGTG
pCMV-Myc-STING-R	tctgtcgacgatatcgaattcTCTGTGTCTGTTGTGGTTATAGTG

F: sense primer; R: antisense primer.

**Table 2:** Correlation of cloning vectors with application systems.

Gene Name	Insert Type	Expression Vector	Application System	Cloning Method
ORF1–ORF73	Viral coding regions	pGBKT7	Y2HS, bait plasmids	One Step Cloning
ORF41	Viral coding regions	pcDNA3.1/His	Co-IP	One Step Cloning
ORF56	Viral coding regions	pcDNA3.1/His	Co-IP	One Step Cloning
IpSTING	Host gene	pGADT7	Y2HS, prey plasmid	One Step Cloning
IpSTING	Host gene	pCMV-C-Myc	Co-IP	One Step Cloning

## Data Availability

The original contributions presented in this study are included in the article/Supplementary Materials. Further inquiries can be directed to the corresponding author.

## Supplementary Materials

The following supporting information can be downloaded at: https://www.mdpi.com/article/10.3390/microorganisms13081780/s1, Figure S1: Nucleotide sequence of IpSTING open reading frame (ORF) and thededuced amino acid sequence.
