# Supplementary material for: Characteristic Analysis of Ictalurus punctatus STING and Screening Validation of Interacting Proteins with Ictalurid herpesvirus 1
Source: Microorganisms. 2025 Jul 30;13(8):1780. doi: 10.3390/microorganisms13081780 (PMC12388441; doi:10.3390/microorganisms13081780)
Supplement: Supplementary file 1 [file microorganisms-13-01780-s001.zip › microorganisms-3735106-SI.pdf]

Characteristic Analysis of *Ictalurus punctatus STING* and Screening Validation of Interacting Proteins with *Ictalurid herpesvirus* 1

Lihui Meng [1,2], Shuxin Li [2], Hongxun Chen [2], Sheng Yuan [2,3] and Zhe Zhao [2,*]

[1] Laboratory of Aquatic Parasitology and Microbial Bioresources, School of Marine Science and Engineering, Qingdao Agricultural University, Qingdao 266237, China; lihuimeng@qau.edu.cn
[2] Jiangsu Province Engineering Research Center for Marine Bio-Resources Sustainable Utilization, College of Oceanography, Hohai University, No. 1 Xikang Road, Nanjing 210098, China; lishuxin@hhu.edu.cn (S.L.); chenhongxun@hhu.edu.cn (H.C.); 210211080007@hhu.edu.cn (S.Y.)
[3] School of Aquatic Science and Technology, Jiangsu Agri-Animal Husbandry Vocational College, Taizhou 225300, China
* Correspondence: zhezhao@hhu.edu.cn

Supplementary Material

```
atg gcg gag gag tgt gtg gtt cca agg cct cgg ggc cat gtg ccg gtg gtg tgt gca agg
 M   A   E   E   C   V   V   P   R   P   R   G   H   V   P   V   V   C   A   R
gtg tta agc agt ctg gca ttg gtg aca gtt tgg ctg ctg tat tcc ggg ttc acg atg tat
 V   L   S   S   L   A   L   V   T   V   W   L   L   Y   S   G   F   T   M   Y
aag tta ata att ctc ata gca caa gtc atg ttc gct ctg agc ctg ggg gtg aca ctg cac
 K   L   I   I   L   I   A   Q   V   M   F   A   L   S   L   G   V   T   L   H
agc ctc tgt ctg ctt act gaa gag tgg ctg ttt cat tca caa cag agg tat ggt ggc agc
 S   L   C   L   L   T   E   E   W   L   F   H   S   Q   Q   R   Y   G   G   S
atc cag cag atg ctc cgg gct tgt ttc agc cgt gct cat gta gta aca atg tgc gtg ggc
 I   Q   Q   M   L   R   A   C   F   S   R   A   H   V   V   T   M   C   V   G
ttt ttg ctg cag ctg gga cag tct gtt tgg aca aat gag aag tgg ctc tta tat gtc tta
 F   L   L   Q   L   G   Q   S   V   W   T   N   E   K   W   L   L   Y   V   L
acc agc act tcc tac ctg ctg ctg aag acc ttc gga tgt ctg ggc cct gta cca gtg gag
 T   S   T   S   Y   L   L   L   K   T   F   G   C   L   G   P   V   P   V   E
atc tca gag gtg tgt gag agc agg aag ctg aat gta gct cat ggc tta gcc tgg tcc tat
 I   S   E   V   C   E   S   R   K   L   N   V   A   H   G   L   A   W   S   Y
tac cta ggt tat ctg aaa ctt gta ata cca gta ctg gag gaa cag ttg agg aaa cac tac
 Y   L   G   Y   L   K   L   V   I   P   V   L   E   E   Q   L   R   K   H   Y
aat cag aac gga gag att ctc cac tct tcc cgg ctg cac atc ctc ctg cct cta agc gca
 N   Q   N   G   E   I   L   H   S   S   R   L   H   I   L   L   P   L   S   A
gtg gtc cca gcc aag gtt gag gaa gag gac cat aac ata tgt ttc cac cag aac ctc ccg
 V   V   P   A   K   V   E   E   E   D   H   N   I   C   F   H   Q   N   L   P
gac ata cag ctc aac cgg gct ggt gtc agg aac aga atc tac aaa aac agc atc tac aaa
 D   I   Q   L   N   R   A   G   V   R   N   R   I   Y   K   N   S   I   Y   K
atc act gac aac caa caa gag ccg tac tac tgt gtg gtg gag tac gcc act cct ctg ctc
 I   T   D   N   Q   Q   E   P   Y   Y   C   V   V   E   Y   A   T   P   L   L
acg ctc tat cag atg tct cag gac agc act gca ggg ttc agc aag cag gac agg aga cag
 T   L   Y   Q   M   S   Q   D   S   T   A   G   F   S   K   Q   D   R   R   Q
cag gtc ctg ctc ttc tac agg act ctc aga caa atc ctg gaa tgt tct cta gag tgt cgc
 Q   V   L   L   F   Y   R   T   L   R   Q   I   L   E   C   S   L   E   C   R
aac cgc tac cac ctt atc ctg ctc gac gac cag cga gct gat ggt gac cct cac tat ctg
 N   R   Y   H   L   I   L   L   D   D   Q   R   A   D   G   D   P   H   Y   L
tcc atg gag atc atc aag cag tta cag cag caa gag cat gag atc ccc atg gac ctg ccg
 S   M   E   I   I   K   Q   L   Q   Q   Q   E   H   E   I   P   M   D   L   P
gaa gaa ctg ccc cag gct gta cca gaa cag ggc gtg aat cat atc cac cag cag gag gag
 E   E   L   P   Q   A   V   P   E   Q   G   V   N   H   I   H   Q   Q   E   E
cca ctc agc tct ctg ccc agc ctg atg att agc gca cca cgt tca ctg aga tca gaa cct
 P   L   S   S   L   P   S   L   M   I   S   A   P   R   S   L   R   S   E   P
gtg gag act aca gac tac acc cac tat aac cac aac aga cac aga
 V   E   T   T   D   Y   T   H   Y   N   H   N   R   H   R
```

Supplementary Figure S1. Nucleotide sequence of IpSTING open reading frame (ORF) and thededuced amino acid sequence.
